# Ultrafine particles and ozone perturb norepinephrine clearance rather than centrally generated sympathetic activity in humans

**DOI:** 10.1038/s41598-019-40343-w

**Published:** 2019-03-06

**Authors:** Karsten Heusser, Jens Tank, Olaf Holz, Marcus May, Julia Brinkmann, Stefan Engeli, André Diedrich, Theodor Framke, Armin Koch, Anika Großhennig, A. H. Jan Danser, Fred C. G. J. Sweep, Christoph Schindler, Katharina Schwarz, Norbert Krug, Jens Jordan, Jens M. Hohlfeld

**Affiliations:** 1 Institute of Aerospace Medicine, German Aerospace Center (DLR), 51147 Cologne, Germany; 2 Fraunhofer Institute for Toxicology and Experimental Medicine (ITEM), 30625 Hannover, Germany; 3 Biomedical Research in Endstage and Obstructive Lung Disease Hannover (BREATH), Member of the German Center for Lung Research, 30625 Hannover, Germany; 4 MHH CRC Core Facility & Centre for Pharmacology and Toxicology, Hannover Medical School, 30625 Hannover, Germany; 5 Institute for Clinical Pharmacology, Hannover Medical School, 30625 Hannover, Germany; 6 Kaufmännische Krankenkasse - KKH, Karl-Wiechert-Allee 61, 30625 Hannover, Germany; 7 Department of Medicine, Division of Clinical Pharmacology, Autonomic Dysfunction Center, Vanderbilt University School of Medicine, Nashville, TN 37232 USA; 8 Institute of Biostatistics, Hannover Medical School, 30625 Hannover, Germany; 9 Division of Vascular Medicine and Pharmacology, Department of Internal Medicine, Erasmus Medical Center, 3015 GE Rotterdam, The Netherlands; 10 Department of Laboratory Medicine, Radboud University Medical Centre, 6500 HB Nijmegen, The Netherlands; 11 Department of Respiratory Medicine, Hannover Medical School, 30625 Hannover, Germany

## Abstract

Cardiovascular risk rapidly increased following exposure to air pollution. Changes in human autonomic regulation have been implicated based on epidemiological associations between exposure estimates and indirect autonomic nervous system measurements. We conducted a mechanistic study to test the hypothesis that, in healthy older individuals, well-defined experimental exposure to ultrafine carbon particles (UFP) increases sympathetic nervous system activity and more so with added ozone (O_3_). Eighteen participants (age >50 years, 6 women) were exposed to filtered air (Air), UFP, and UFP + O_3_ combination for 3 hours during intermittent bicycle ergometer training in a randomized, crossover, double-blind fashion. Two hours following exposure, respiration, electrocardiogram, blood pressure, and muscle sympathetic nerve activity (MSNA) were recorded at supine rest, during deep breathing, and during a Valsalva manoeuvre. Catechols and inflammatory marker levels were measured in venous blood samples. Induced sputum was obtained 3.5 h after exposure. Combined exposure to UFP + O_3_ but not UFP alone, caused a significant increase in sputum neutrophils and circulating leucocytes. Norepinephrine was modestly increased while the ratio between plasma dihydroxyphenylglycol (DHPG) and norepinephrine levels, a marker for norepinephrine clearance, was reduced with UFP + O_3_. Resting MSNA was not different (47 ± 12 with Air, 47 ± 14 with UFP, and 45 ± 14 bursts/min with UFP + O_3_). Indices of parasympathetic heart rate control were unaffected by experimental air pollution. Our study suggests that combined exposure to modest UFP and O_3_ levels increases peripheral norepinephrine availability through decreased clearance rather than changes in central autonomic activity. Pulmonary inflammatory response may have perturbed pulmonary endothelial norepinephrine clearance.

## Introduction

The World Health Organization estimated that air pollution is the 13th leading cause of mortality worldwide^1^. A large proportion of the excess mortality can be attributed to cardiovascular causes^2^. The risk for acute myocardial infarction was increased two and 24 hours following increased fine particle exposure^3^. Ventricular arrhythmias were also more likely when patients were subject to short-term exposure to fine particles^4^. During an air pollution episode, average resting heart rate and blood pressure were modestly increased. The odds ratio of having hypertensive blood pressure readings was 1.63^5,6^. The rapid increase in blood pressure, heart rate, and cardiovascular risk suggests autonomic nervous system involvement with augmented adrenergic drive and parasympathetic withdrawal. Indeed, for each 1 mg/m^3^ increment in workplace exposure to fine particles with ≤2.5 µm mean aerodynamic diameter (PM2.5), heart rate variability in 24-hour electrocardiograms decreased 2.66%^7^. Heart rate variability decreased within two hours^8^ and remained suppressed the following day^9^. Experimental exposure to mixed coarse and fine particles elicited similar responses^10^. Fine particle exposure was also pressor in rats^11,12^. On the other hand, an air-filtering device was reported to attenuate plasma norepinephrine concentrations in humans^13^. Older persons may be more susceptible to air pollution because of detrimental effects on autonomic cardiovascular control as opposed to young subjects^14^. Indeed, particulate matter and O_3_ have been shown to decrease heart rate variability in this population^15^. Greater susceptibility may also arise from the ability of nanoparticles to cause localized accumulations after translocation from airways and lungs into the brain via neuronal and circulatory pathways^16^. Furthermore, the adverse effects of air pollution may have greater impact on the health status in the elderly^17^. Ultrafine particles with ≤0.1 µm aerodynamic diameter appear to be particularly harmful because they escape broncho-mucociliary clearance and reach alveoli^18^. Epidemiological studies showed associations between ultrafine carbon particle exposure and cardiopulmonary morbidity and mortality which may operate as a universal carrier for various chemicals^19^. Ozone (O_3_) is another pollutant contributing to adverse cardiovascular effects^20–22^. Airway inflammation is augmented with concomitant exposure to carbon particles and O_3_^23^.

So far, much of the evidence linking air pollutant exposure with changes in human cardiovascular autonomic regulation relied on epidemiological studies, exposure estimates, and indirect autonomic nervous system measurements. Moreover, mechanism-oriented studies are rarely conducted in older persons who are more susceptible to air pollution. Therefore, we conducted a study in healthy individuals >50 years, to test the hypotheses that short-term experimental exposure to UFP changes the balance between sympathetic and parasympathetic cardiovascular control towards sympathetic predominance. We reasoned that combined exposure to particles and O_3_ would have an even greater effect. Our findings suggest that short-term experimental air pollution augments peripheral norepinephrine availability through changes in peripheral norepinephrine clearance rather than increased efferent sympathetic nerve traffic.

## Methods

### Subjects

We screened 57 healthy subjects >50 years of age. Eight postmenopausal women and 17 men met all inclusion and exclusion criteria and entered the study between September 2013 and June 2015. Smokers and individuals with clinically apparent cardiovascular and/or pulmonary diseases were excluded. Specifically, we excluded patients with asthma of any severity while atopy or atopic rhinitis was not an exclusion criterion. We included 1 subject with asymptomatic atopy and 2 subjects with mild or sporadic rhinitis symptoms during their respective pollen season. Both had their visits outside of their respective season. We also excluded individuals on medications strongly affecting autonomic nervous system function, e. g. norepinephrine reuptake inhibitors, whereas stable medication with slight to moderate autonomic effects was tolerated. We did not select subjects with regard to their domestic or workplace air pollution.

Twenty participants (6 women and 14 men) completed all three study visits with successful microneurography recordings. Two participants had lost substantial amounts of body weight during the study even though we had advised all participants not to change their exercise and dietary habits. Because both had begun an intense lifestyle intervention including regular physical training, they were excluded before the study was unblinded leaving 18 participants in the primary analysis set. The CONSORT diagram for the trial is shown in Supplementary Fig. S1. Characteristics of the study population are given in Table 1.

**Table 1:** Subject characteristics (mean ± SD).

Parameter				
Number			18	
Male/female		12	/	6
Age	[years]	59.2	±	7.0
Body mass	[kg]	85.6	±	18.7
Height	[cm]	176	±	9
BMI	[kg/m²]	27.3	±	4.7

BMI: Body mass index.

### Study Design and Protocol

The ethics committee of Hannover Medical School approved our study and all participants gave written informed consent before enrolment. The methods were carried out in accordance with the relevant guidelines and regulations. The study was registered at clinicaltrials.gov (registration number: NCT01914783, posted: 2013-08-02). In this randomized, sham-controlled, double-blind, three-period, three-sequence crossover study participants were exposed to Air (placebo), UFP, and the combination UFP + O_3_. Washout periods between exposures were at least eight weeks. Data were collected at the Fraunhofer Institute for Toxicology and Experimental Medicine (Hannover ITEM) and at the Clinical Research Centre of Hannover Medical School.

### Exposure

We exposed subjects to Air, UFP, or UFP + O_3_, in a dedicated exposure chamber equipped with a bicycle ergometer. UFP (50 μg/m³) and O_3_ (250 ppb) generation are described in Supplementary Methods^24,25^. Detailed justification for UFP and O_3_ dosage is given in Supplementary Methods. In brief, the O_3_ concentration used in our study exceeds the 1 hour alarm level in Germany about 2 fold; the UFP exposure is in the range of current PM10 and PM2.5 threshold levels.

At experimental visits, participants were continuously exposed for 3 h (Supplementary Fig. S2). During the exposure period subjects underwent alternating 15-min cycles of rest and exercise at previously determined workload (Supplementary Methods). The approach has been used in numerous challenge studies worldwide and guarantees a sufficient inflammatory response^26^. We monitored ECG and oxygen saturation continuously and measured brachial blood pressure twice at the end of each cycle. Following exposure, subjects ingested a standardized light lunch. Then, they were instrumented for cardiovascular and autonomic measurements which started approximately 1.5 h after the exposure. Finally, ~3.5 h after the end of the exposure, participants were subjected to sputum induction (Supplementary Methods) before they went home. The inflammatory response after a 3-h exposure to 250 ppb O_3_ can already be detected 1 h after exposure and lasts at least for 24 h. The chosen time window for autonomic, cardiovascular, and sputum measurements corresponds to the inflammatory response^27^.

### Measurements

Subjects were instrumented in the supine position in a quiet laboratory at an ambient temperature of 22–23 °C. We inserted an antecubital venous catheter and continuously recorded respiration, ECG, thoracic impedance (Cardioscreen, Medis GmbH, Ilmenau, Germany), and beat-by-beat finger blood pressure (Finometer, FMS, Arnhem, The Netherlands). We also measured brachial blood pressure with an automated oscillometric device (Dinamap, GE Medical Systems, Milwaukee, WI, USA) and cardiac output by inert gas rebreathing (Innocor, Innovision, Odense, Denmark). We recorded postganglionic, multiunit muscle sympathetic nerve activity (MSNA) from within the peroneal nerve. After achieving stable resting baseline, respiration, blood pressure, heart rate, and MSNA were recorded for 10 minutes followed by cardiac output measurements and venous sampling for catecholamine, renin, and dihydroxyphenylglycol (DHPG) determination (Supplementary Methods)^28–30^. Then, subjects underwent controlled slow breathing (6 breaths per minute) and a Valsalva manoeuvre for hemodynamic and autonomic assessment. Thereafter, we obtained venous blood samples for inflammatory marker quantification. Finally, we used procedures for sputum induction and flow cytometry of sputum cells as described previously^31^. We obtained estimates of heart rate and systolic blood pressure variability from 5-min supine rest recordings. Spontaneous cardiac baroreflex sensitivity was calculated as the slope of the linear regression between instantaneous systolic blood pressure and subsequent R-R intervals using the sequence method^32^ or by cross-spectral transfer function analysis in the low-frequency range between 0.05 and 0.15 Hz (Supplementary Methods).

### Statistics

We planned the study to test the following hypotheses: In healthy older subjects, experimental exposure to carbon black UFP increases sympathetic nervous system activity compared with filtered Air sham exposure (primary hypothesis). Moreover, combined UFP + O_3_ exposure increases sympathetic nervous system activity more than UFP exposure alone (secondary hypothesis). The primary endpoint of the study was 5-min resting MSNA burst frequency [bursts/min]. Secondary endpoints were resting MSNA burst incidence [bursts/100 heart beats], which corrects MSNA for heart rate, and total MSNA [arbitrary units], which takes into account burst strength. We also explored arterial blood pressure, heart rate, cardiac output, blood pressure and heart rate variability, baroreflex sensitivity, plasma norepinephrine, DHPG, and renin concentrations, and inflammation parameters.

Analysis of the primary endpoint was performed using an analysis of variance (ANOVA) model that allows for the adjustment for period effects according to the Hills-Armitage approach using a fixed effects model (Supplementary Methods)^33^. A closed testing procedure was used to preserve a two-sided type-I error of 0.05: The overall test in the ANOVA had to have a *P* value < 0.05 to proceed to pairwise comparisons between exposure groups. All remaining parameters were analysed by repeated measures ANOVA without adjustment for multiple comparisons. Exposure information is expressed as median ± interquartile range (IQR); physiologic data are expressed as mean ± SD. For primary endpoint contrasts confidence intervals [CI] are also provided.

## Results

### Experimental exposure to UFP and O_3_

During exposure with Air, UFP, and UFP + O_3_ particle mass concentrations were 19.9 ± 10.2, 68.5 ± 13.7, and 69.1 ± 19.8 µg/m³, respectively. UFP diameters were 50.6 ± 1.9 (UFP alone) and 47.5 ± 3.6 nm (UFP + O_3_) with a mean geometric standard deviation in particle size of 1.7 nm. The median O_3_ concentration during UFP + O_3_ exposure was 249.5 ± 3.7 ppb. Median relative humidity was 42.1 ± 11.6, 36.3 ± 10.4, and 41.5 ± 11.1% with median temperatures of 22.6 ± 0.3, 22.7 ± 0.2, and 22.6 ± 0.2 °C during exposure to Air, UFP, and UFP + O_3_, respectively.

### Combined exposure to UFP + O_3_ elicits local pulmonary inflammation affecting pulmonary function

**Figure 1:**
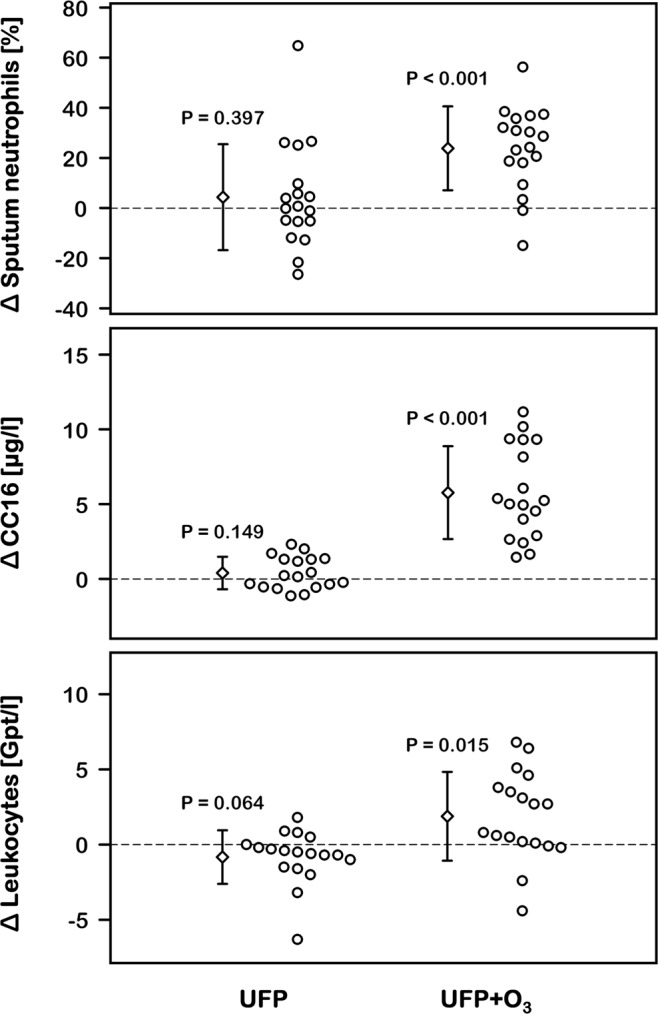
Inflammatory markers (n = 18). The plots show individual differences of inflammatory markers with ultrafine particles or ultrafine particles + ozone compared with filtered air. Only combined ultrafine particles + ozone exposure caused an increase in these markers. Serum malondialdehyde and high-sensitivity C-reactive protein did not change with exposure and have been omitted. Upper panel: Neutrophils in sputum that has been obtained ~3.5 h after the end of exposure. Lower panels: Club cell protein levels (CC16) and leukocytes in blood samples that have been obtained ~2.5 h after the end of exposure.

UFP exposure alone did not significantly change inflammatory markers. In contrast, the percentage of neutrophils in induced sputum (P < 0.001), serum club cell protein (CC16) levels (P < 0.001), and blood leukocytes (P < 0.001) were significantly increased following combined UFP + O_3_ exposure (Fig. 1, Supplementary Table S1). Serum myeloperoxidase, malondialdehyde, and high-sensitivity C-reactive protein did not change. Localized pulmonary inflammation with UFP + O_3_ was associated with modest impairments in lung function (Supplementary Table S1). Over 3 h of intermittent exercise in Air we observed increases in forced vital capacity (ΔFVC: 191 ± 180 ml, P < 0.001) and forced expiratory volume in one second (ΔFEV1: 102 ± 89 ml, P < 0.001). Such increases were lacking with combined UFP + O_3_ exposure (ΔFVC: 8 ± 343 ml, P = 0.918; ΔFEV1: −57 ± 210 ml, P = 0.262). During the microneurographic measurements approximately 2 h after combined UFP + O_3_ exposure, respiratory rate tended to be slightly elevated (P = 0.085, Table 2). These findings are in line with the idea that acute O_3_–induced lung function changes are dominated by involuntary inhibition of inspiration^34^. Lung function was unchanged with UFP exposure alone suggesting that both pollutants may interact in terms of pulmonary inflammation and that inflammation is required to acutely perturb pulmonary function. Nevertheless, exposures were well tolerated and we did not observe clinically relevant changes in lung function, reduced oxygen saturation, or serious adverse events.

**Table 2:** Resting hemodynamics and respiration during and after exposure (mean ± SD).

Parameter		Air			UFP			UFP + O_3_			*P* value
*During exposure (rest between exercise bouts)*											
SBP	[mm Hg]	118	±	12	119	±	11	120	±	11	0.481
DBP	[mm Hg]	72	±	8	73	±	7	73	±	7	0.417
HR	[bpm]	75	±	11	74	±	12	73	±	12	0.644
*After exposure (supine rest during microneurography)*											
SBP	[mm Hg]	125	±	16	126	±	17	126	±	16	0.760
DBP	[mm Hg]	74	±	10	73	±	9	73	±	9	0.676
HR	[bpm]	73	±	9	74	±	11	73	±	12	0.802
CO	[l/min]	6.70	±	1.01	6.93	±	0.88	6.82	±	1.14	0.771
TPR	[dyn × s/cm^−5^]	1000	±	183	986	±	221	991	±	218	0.954
f_Resp_	[breaths/min]	15.0	±	2.6	15.4	±	2.9	16.2	±	3.1	0.085

HR: Heart rate, SBP: Systolic blood pressure, DBP: Diastolic blood pressure, CO: Cardiac output, TPR: Total peripheral resistance, f_Resp_: Respiratory rate.

### Evidence for increased systemic norepinephrine availability

**Figure 2:**
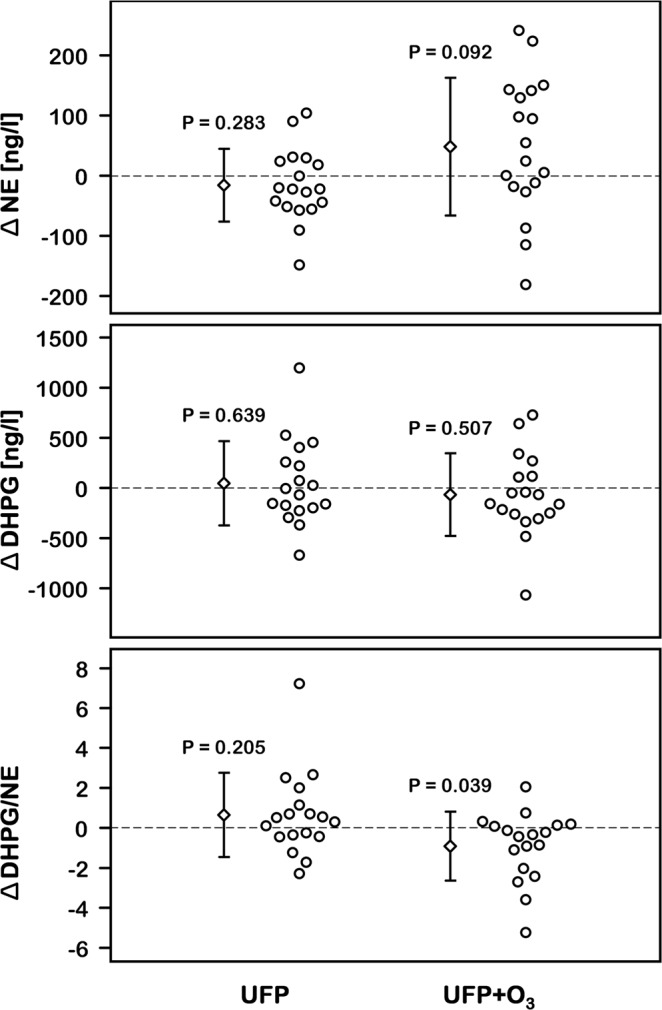
Catechols (n = 18). The plots show individual differences of catechols with ultrafine particles (UFP) or UFP + ozone (O_3_) compared with filtered air. Assuming the null hypothesis, the mean of the data points would fall on the zero line (dashed line). Combined exposure to UFP + O_3_ tended to increase norepinephrine plasma levels (NE, upper panel). The ratio between dihydroxyphenylglycol (DHPG, middle panel) and NE may serve as biochemical indicator for norepinephrine reuptake (lower panel). Its decrease could explain the increase in plasma NE.

We conducted a detailed biochemical catechol analysis to gauge influences of experimental air pollution on peripheral catecholamine availability and metabolism. UFP alone did not elicit changes in venous plasma epinephrine or norepinephrine concentrations. However, following UFP + O_3_, we observed a significant increase in plasma norepinephrine concentrations (P = 0.020, Table 3) while plasma epinephrine did not change (P = 0.254). The half-life of catecholamines in plasma is less than 2 mins. Therefore, increased norepinephrine levels more than 1.5 h after the exposure cannot be explained by sympathetic activation during exposure. Much of the released norepinephrine is taken up through the neuronal norepinephrine transporter (uptake 1) and subsequently metabolized via monoamine oxidases to dihydroxyphenylglycol (DHPG). Therefore, plasma DHPG and the DHPG to norepinephrine ratio can be utilized to assess norepinephrine clearance mechanisms. Following UFP + O_3_, we observed a reduction in the DHPG to norepinephrine ratio (P = 0.016, Table 3, Fig. 2). The finding suggests that the increase in venous plasma norepinephrine following UFP + O_3_ is caused at least in part by reduced peripheral norepinephrine uptake and metabolism.

**Table 3:** Autonomic nervous system after exposure (mean ± SD).

Parameter	Air			UFP			UFP + O_3_			*P* value
*MSNA (burst frequency*, *burst incidence*, *total activity)*										
[bursts/min]	47.2	±	12.3	46.9	±	14.5	44.8	±	14.0	0.633
[bursts/100 heart beats]	64.6	±	13.5	63.2	±	15.3	61.7	±	17.1	0.672
[au]	2.97	±	2.16	2.75	±	1.46	2.75	±	1.47	0.827
*Catechol derivatives*										
Dopamine [ng/l]	17.3	±	12.9	15.5	±	8.5	18.7	±	8.3	0.256
Epinephrine [ng/l]	18.3	±	11.7	17.4	±	12.0	21.0	±	14.3	0.254
NE [ng/l]	356	±	125	340	±	146	404	±	124	0.020*
NE/MSNA [ng/l/au]	164	±	89	150	±	84	190	±	106	0.278
DHPG [ng/l]	1865	±	443	1912	±	576	1799	±	373	0.551
DHPG/NE	5.56	±	1.64	6.21	±	2.60	4.66	±	1.17	0.016*
*RR interval variability*										
LF [ms²]	425	±	545	524	±	675	547	±	991	0.626
HF [ms²]	166	±	287	183	±	305	241	±	536	0.355
TP [ms²]	1132	±	1194	1416	±	1512	1147	±	1585	0.171
LF/HF ratio	3.45	±	2.88	3.89	±	2.79	3.39	±	2.72	0.760
SD [ms]	33.1	±	15.2	38.2	±	19.6	34.3	±	21.2	0.258
RMSSD [ms]	21.6	±	16.2	22.4	±	16.6	25.8	±	26.0	0.385
*SBP variability*										
LF [mm Hg²]	16.2	±	16.6	14.8	±	10.4	14.3	±	8.6	0.858
*Baroreflex sensitivity (baroreflex gain) [ms/mm Hg]*										
Down sequences	7.0	±	3.3	6.1	±	2.8	6.2	±	3.5	0.283
Up sequences	6.8	±	3.7	6.1	±	2.8	6.9	±	4.9	0.514
Cross-spectral analysis	5.4	±	2.4	5.5	±	2.8	6.2	±	4.1	0.501

MSNA: Muscle sympathetic nerve activity, au: Total MSNA in arbitrary units per minute, NE: Plasma norepinephrine, DHPG: Dihydroxyphenylglycol, *Significant difference between UFP and UFP + O_3_ (see also Fig. 2 for differences vs Air). VLF: Very low frequency band, LF: Low frequency band, HF: High frequency band, TP: Total power, SD: Standard deviation of RR intervals, RMSSD: Root mean square of successive RR interval differences, SBP: Systolic blood pressure.

### Changes in norepinephrine availability are not explained by altered central autonomic nervous system regulation

**Figure 3:**
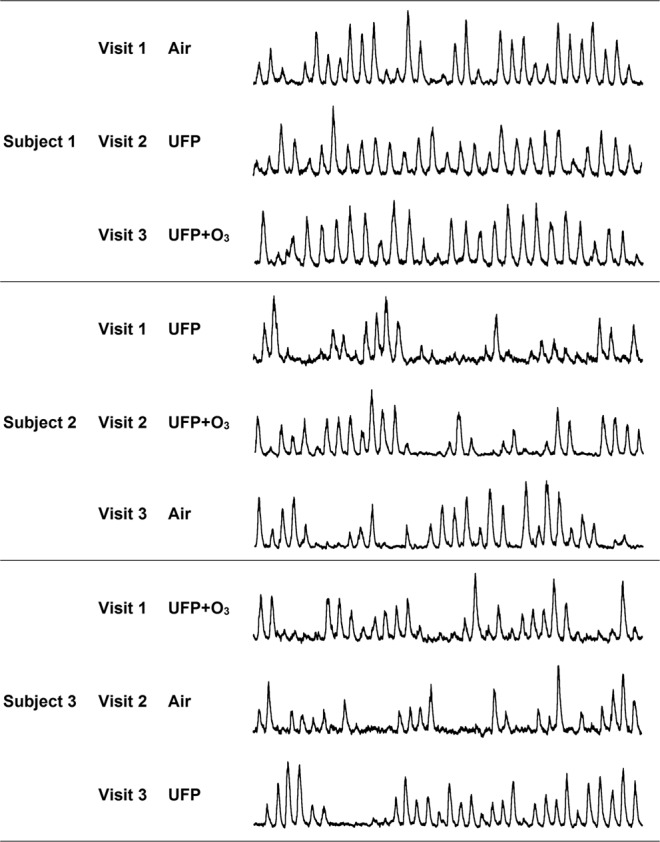
Original muscle sympathetic nerve activity (MSNA) recordings. Representative MSNA recordings (30-s segments) in 3 subjects with differing exposure sequences. There is no indication of sympathetic activation during exposure to ultrafine particles with or without additional ozone.

Muscle sympathetic nerve activity (MSNA) measurements through microneurography are considered the Gold standard to assess vasoconstrictor sympathetic nerve traffic in human beings. Figure 3 illustrates representative MSNA recordings during the three-sequence crossover study for three subjects with different exposure sequences. MSNA and neurohumoral data are presented in Table 3, Fig. 2, and in Supplementary Table S2. We did not observe clinically relevant differences in MSNA burst frequency between exposures. The overall test yielded a P value of 0.633, and all pairwise comparisons expressed in bursts/min were UFP + O_3_ minus UFP: −2.3, 95% CI [−8.5, 3.9], P = 0.458; Air minus UFP: 0.5, 95% CI [−5.8, 6.7], P = 0.883; UFP + O_3_ minus Air: −2.8, 95% CI [−9.0, 3.5], P = 0.376. Moreover, also MSNA burst incidence and MSNA total activity did not differ between exposures. Overall, these observations exclude a major change in centrally generated sympathetic activity with experimental air pollution. The conclusion is supported by the observation that plasma renin concentrations, which respond to changes in renal sympathetic drive, did not increase with experimental air pollution (22.5 ± 42.1, 16.5 ± 21.8, and 15.3 ± 12.0 ng/l following exposure to Air, UFP, and UFP + O_3_, respectively P = 0.425).

Next, we computed indirect measures for autonomic cardiovascular control that respond to changes in central nervous sympathetic or parasympathetic control (Table 3). Systolic blood pressure variabilities were comparable following exposure to Air, UFP, or UFP + O_3_. RR interval variability and cardiac baroreflex gain, which are related to cardiac vagal activity, did not differ between interventions. Likewise, maximum sympathetic response during Valsalva phase IIb was similar as was respiratory sinus arrhythmia with deep breathing and Valsalva ratio as indices of parasympathetic heart rate control. Thus, modest experimental air pollution while producing pulmonary inflammation and changes in lung function is not sufficient to perturb central sympathetic or parasympathetic cardiovascular control mechanisms. Since sympathetic nerve traffic and the relation between norepinephrine and MSNA were unchanged, the increase in norepinephrine with UFP + O_3_ cannot be explained by augmented centrally generated sympathetic outflow.

### Altered norepinephrine availability does not translate into changes in hemodynamic measurements

Resting hemodynamic and respiration data obtained during microneurography measurements are provided in Table 2. Hemodynamics were similar between study days. We reasoned that influences of experimental air pollution on hemodynamics could be unmasked during states of raised sympathetic activity. Therefore, we also assessed hemodynamic responses during exercise in the exposure chamber (see Supplementary Fig. S3 for individual data). Heart rate and systolic blood pressure increased similarly with exercise on the three study days (Air: 41.9 ± 11.4, UFP: 37.2 ± 14.3, UFP + O_3_: 38.1 ± 15.8 bpm, *P* = 0.350; Air: 29.5 ± 12.9, UFP: 26.7 ± 11.6, UFP + O_3_: 27.0 ± 13.9 mm Hg, *P* = 0.488). Diastolic blood pressure increased less with UFP vs. UFP + O_3_ (Air: 2.7 ± 7.5, UFP: 0.4 ± 4.7, UFP + O_3_: 4.5 ± 6.9 mm Hg, *P* = 0.028). Resting hemodynamics between the 15-min exercise bouts were similar during all exposures (Table 2). Thus, in otherwise healthy older persons, experimental air pollution while eliciting pulmonary inflammation and reduced norepinephrine turnover does not produce major changes in blood pressure and heart rate control at rest or during physical exercise.

## Discussion

Our study is the first examining the effects of rigorously controlled short-term experimental exposure to UFP with and without O_3_ on directly measured sympathetic activity. The main finding is that the exposure did not elicit clinically relevant changes in sympathetic activity at rest or during sympathetic stimulation. However, we observed an about 13% increase in venous norepinephrine concentrations with reduction in the DHPG to norepinephrine ratio following combined UFP + O_3_ exposure. By comparison, smoking 20 cigarettes per day over one week increased plasma norepinephrine approximately 10% compared with air inhalation^35^. Our study suggests diminished catecholamine clearance, a hitherto unknown mechanism through which environmental pollutants may perturb human adrenergic responses. In healthy subjects small amounts of pressor compounds do not translate into blood pressure increases as they are effectively balanced by counterregulatory responses, such as baroreflexes. In diseased subjects with elevated sympathetic activity and diminished baroreflex function, however, reduced norepinephrine reuptake may have detrimental effects.

Highly controlled exposure to environmental pollutants in a rigorously designed double-blind and crossover fashion is a particular strength of our study. Measured exposures to UFP and O_3_ were rather stable with little deviation from planned experimental conditions. We applied UFP and O_3_ concentrations that are known to elicit biological responses in human subjects. Carbon UFP inhalation in concentrations applied in our study transiently activated platelets in patients with type-2 diabetes mellitus^36^. Unlike UFP exposure, which is known to have a limited effect on human airway inflammation, addition of O_3_ elicited a robust inflammatory response. In fact, sputum neutrophil counts and circulating inflammatory markers increased sharply. In induced sputum, the magnitude of the inflammatory response to combined UFP + O_3_ exposure was similar to studies applying O_3_ only^37^. Bronchial biopsies taken following acute O_3_ exposure in healthy subjects showed increased percentage of neutrophils and total protein concentration^38,39^.

Combining microneurography with hemodynamic measurements, plasma catecholamine determination, and heart rate as well as blood pressure variability analysis provided comprehensive insight in cardiovascular autonomic responses to experimental air pollution. It has been speculated that environmental pollutants may engage afferent neural pathways in the lung or elsewhere in the body, thereby changing autonomic balance towards sympathetic predominance. In fact, O_3_ responsive bronchial C fibres have been demonstrated in anesthetized and artificially ventilated dogs^40^. Due to their size, UFP may pass the alveolo-capillary barrier^41^ and enter the brain^42^ where they could directly affect autonomic circuits. Yet, we did not observe clinically relevant changes in MSNA at rest, during deep breathing, or during baroreflex-mediated sympathetic activation elicited by the Valsalva manoeuvre. Blood pressure and heart rate did not respond either. Furthermore, blood pressure and heart rate recordings during exposure did not differ between interventions. Our findings challenge the idea that UFP with or without O_3_ elicit substantial changes in centrally generated sympathetic activity or parasympathetic heart rate control.

The plasma norepinephrine increase following UFP + O_3_ exposure cannot be explained by increased central sympathetic drive because MSNA did not change. Dissociation between circulating norepinephrine and MSNA could result from altered coupling between electrical nerve activity and transmitter release or changed norepinephrine uptake/metabolism. Approximately 80–90% of the released norepinephrine is taken up again by the norepinephrine transporter and either repackaged or enzymatically degraded by monoamine oxidases to DHPG. Therefore, the DHPG to norepinephrine ratio has been proven useful as biochemical marker for neural norepinephrine uptake and metabolism^43^. Indeed, genetic norepinephrine transporter deficiency^44^ and pharmacological norepinephrine transporter inhibition^45–47^ feature reductions in the ratio between plasma DHPG and norepinephrine. The observation that the ratio was reduced following exposure to UFP + O_3_ is consistent with reduction in norepinephrine uptake and metabolism through this pathway. Besides their expression on adrenergic neurons, norepinephrine transporters are also present on lung endothelial cells^48,49^, and contribute to catecholamine clearance in newborn lambs, in dogs, and in human subjects^50–52^. We cannot differentiate individual contributions of lung endothelium and adrenergic neurons to the change in the DHPG/norepinephrine ratio. Particulate matter inhalation causes endothelial injury, reflected by circulating endothelial microparticles derived from apoptosis^53^. We speculate that pulmonary endothelial inflammation could affect endothelial catecholamine clearance through the norepinephrine transporter. If so, reduced clearance could augment catecholamine concentrations entering the coronary circulation, particularly during profound sympathoadrenal activation. We cannot exclude that other local or systemic changes, that are affected by UFP + O_3_ exposure, contributed to the response. The cellular regulation of the norepinephrine transporter has been studied in great detail^54^. For example, protein kinase-C, which is targeted by multiple neurotransmitters and hormones, affects norepinephrine transporter trafficking. Yet, whether and how these mechanisms contribute to norepinephrine uptake in human beings is largely unknown.

## Limitations

Our study was sufficiently powered to detect clinically relevant changes in MSNA. We cannot exclude subtle changes in cardiovascular autonomic control that may be relevant on the population level. Yet, MSNA was numerically lower following UFP exposures compared to filtered air. Furthermore, environmental fine particles contain minerals, metals, road dust, combustion residues, SO_2_ and NO_X_ from fires and engine exhaust among others. Particle composition depends on sources, site, season, and weather conditions^55^. Potential interactions between components of airborne substance mixtures on human health have been poorly investigated^56^. Observations on carbon UFP cannot be simply extrapolated to environmental UFP exposure. In any event, exposure to particles sampled at different locations produced differential cardiovascular responses in human subjects^57^. We did not obtain a full dose-response relationship between exposure and cardiovascular autonomic regulation. We used a short-term exposure model whereas typical ambient air pollution periods are of longer duration and often repetitive. Yet, even two-hour exposure may trigger cardiovascular events^3,8^. Our idea was to assess autonomic function using microneurography and plasma sampling when the inflammatory process is fully developed. However, we cannot rule out that there is inflammation-independent sympathetic excitation that would have been detected only during or shortly after the exposure. Finally, we studied older otherwise healthy individuals and, therefore, cannot exclude more marked responses in patients with established cardiovascular or pulmonary disease.

## Conclusions

The epidemiological evidence implicating environmental pollutants in cardiovascular morbidity and mortality is compelling. Since a larger proportion of the world population will live in urban environments in the future, the relevance of these mechanisms for cardiovascular health may increase further. Yet, epidemiological studies have their limitations in discerning the contribution of individual pollutants on cardiovascular health and may not suffice to design targeted interventions. The fact that air pollution and environmental noise^58^, which commonly occur together, are both associated with increased cardiovascular risk is a prime example. Our observations suggest that exposure to well-defined pollutants in a rigorously controlled environment combined with high-fidelity human phenotyping is suitable to confirm or to exclude hypotheses generated in large-scale epidemiological studies and to gain mechanistic insight. While acute UFP exposure with or without O_3_ did not alter central sympathetic or parasympathetic activity, we observed subtle changes in peripheral catechols with combined exposure to UFP and O_3_ consistent with impaired norepinephrine uptake and metabolism. We suggest that the pulmonary inflammatory response may have perturbed pulmonary endothelial norepinephrine clearance.

## Supplementary information

SI

## Data Availability

The datasets generated during and/or analysed during the current study are available from the corresponding author on reasonable request.
