# Supplementary material for: Ultrafine particles and ozone perturb norepinephrine clearance rather than centrally generated sympathetic activity in humans
Source: Sci Rep. 2019 Mar 6;9:3641. doi: 10.1038/s41598-019-40343-w (PMC6403347; doi:10.1038/s41598-019-40343-w)
Supplement: Supplementary file 1 — SI [file 41598_2019_40343_MOESM1_ESM.docx]

Supplementary Information

Ultrafine particles and ozone perturb norepinephrine clearance
rather than centrally generated sympathetic activity in humans

Karsten Heusser, Jens Tank, Olaf Holz, Marcus May, Julia Brinkmann, Stefan Engeli, André Diedrich, Theodor Framke, Armin Koch, Anika Großhennig, A. H. Jan Danser, Fred C. G. J. Sweep, Christoph Schindler, Katharina Schwarz, Norbert Krug,
Jens Jordan, and Jens M. Hohlfeld

# Methods

## Subjects


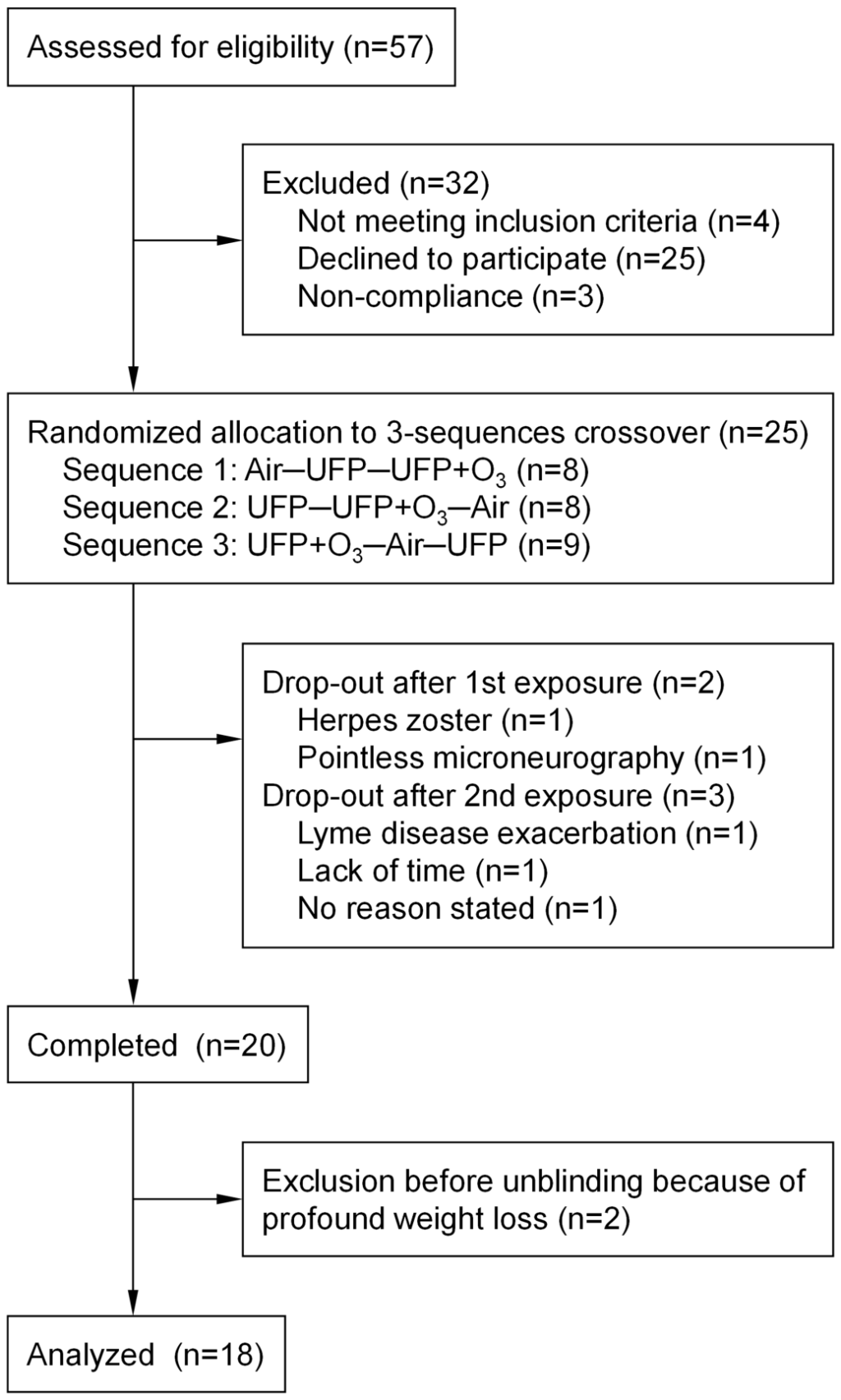


1. CONSORT flow diagram of subject enrolment, allocation, and analysis.

## Exercise testing to determine individual workloads

At the screening visit, participants were submitted to an exercise test to determine the individual workload required to increase ventilation to approximately 20 l/min/m² body surface. Exercise testing was conducted on a bicycle ergometer with adjustable workload. After a two-min baseline, workload was increased stepwise (10 to 20 W). We asked participants to pedal at a constant speed of 60 rpm. Every two minutes, before further load adjustment, minute ventilation was assessed using a spirometer (SpiroPro: specifically programmed for exhaled breath condensate measurements, CareFusion, Germany). As soon as participants attained target ventilation, the corresponding workload was maintained for another five minutes. During exercise, ECG was continuously monitored and the test was discontinued if the patient asked to do so or if the investigator considered it necessary.

## Experimental exposure visit schedule


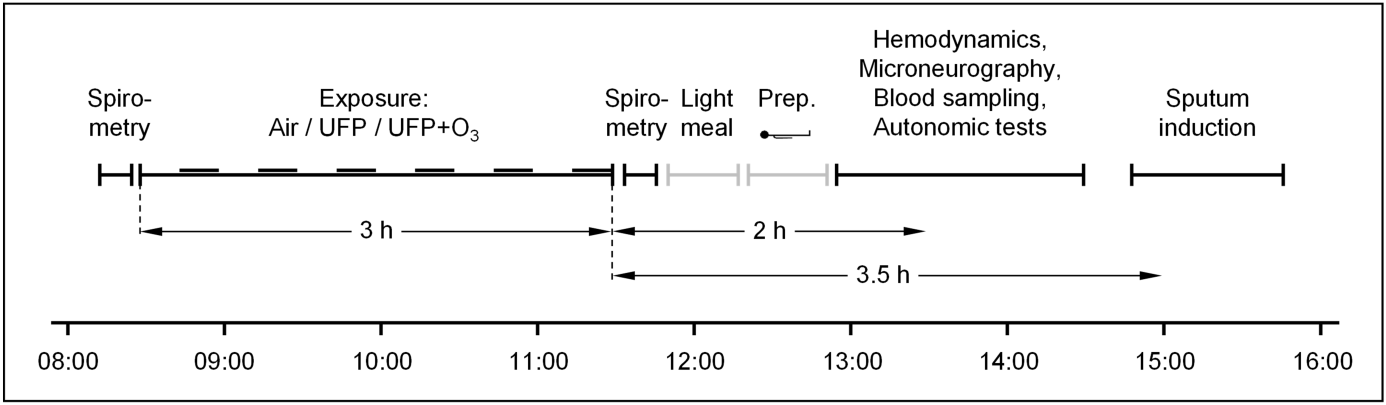


1. Visit schedule. In the morning hours, participants were continuously exposed to clean air, ultrafine particles, or ultrafine particles and ozone for 3 h. During the exposure period participants followed an alternating 15-min schedule of rest and exercise. After showering and a light lunch they underwent sympathetic microneurography and hemodynamic measurements while resting in supine position and during autonomic function testing. At the end of the study visit, sputum samples were obtained. During periods drawn in black we collected data and samples.

## Exposure

In Germany the 1 hour mean warning value for ozone is 90 ppb (1 hour mean alarm level = 120 ppb), and currently the highest values detected in Germany are below 150 ppb. The threshold values for PM10 and PM2.5 are 40 and 25 µg/m³, respectively. There are no threshold levels for ambient UFP yet. In 2011 the US Occupational Health Inst. NIOSH suggested to use 2.4 mg/m³ for Titanium dioxide (>0.1 µm) and 0.3 mg/m³ for smaller UFP at workplaces. A limit for particle concentration of 20.000 particles/cm³ is currently discussed. The ozone concentration used in our study exceeds the 1 hour alarm level in Germany about 2-fold. 250 ppb have been used in numerous studies over exposure times of 3 hours and are known to result in a transient neutrophilic airway inflammation. Based on our experience there are a few ozone sensitive subjects, which respond at this concentration with cough and larger decline in lung function. Due to this, we monitored lung function during the exposure.

The UFP exposure with 50 µg/m³ is in the range of current PM10 and PM2.5 threshold levels but exceeds the discussed thresholds for UFP. We designed our trial based on former experience with carbon black particle exposures.^1–3^ 50 µg/m³ carbon UFP exposures have been previously performed in younger healthy subjects.^1^ UFP (carbon black aerosol) were generated by a commercially available electric spark generator (Type GFG 1000, Palas, Germany) using highly purified elemental graphite electrodes in an argon atmosphere as described previously.^4^ Production and volume flow rates into the challenge chamber were adjusted to attain highly reproducible concentrations of 50 μg/m³. For each exposure, the particle mass concentration in the exposure chamber was measured by gravimetric analysis of filter samples and the corresponding cumulative sample volume during the entire exposure period. In addition, on-line monitoring of the number size distribution was carried out using a scanning mobility particle sizer spectrometer. Particles detected during Air exposure are primarily derived from clothing abrasion (background particles) and need to be subtracted from the particles mass concentrations during UFP exposures. Typically, these particles are in the micrometre range and not inhalable. In comparison with UFP and UFP+O_3_ their number is negligible. Nevertheless, to minimize the influence of the background particles, subjects were asked to wear the same clothing during the three exposure days.

We generated O_3_ from medical oxygen using a commercial device (COM-ADM, ANSEROS GmbH Tübingen, Germany) producing high purity ozone mass flux. The 250 ppb (parts per billion = parts/10^9^) target concentration of ambient O_3_^5^ was continuously monitored by two independent analysers (Ozomat MP, ANSEROS GmbH Tübingen, and 400A, MLU-Messtechnik für Luft und Umwelt GmbH Essen, Germany).

## Microneurography and Data Analysis

We obtained muscle sympathetic nerve activity from the peroneal nerve in the popliteal space using unipolar tungsten microneurography needles (UNA30F2S, Frederick Haer & Co., Bowdoinham, ME; USA). Correct microelectrode position in a muscle fascicle containing efferent vasoconstrictor C-fibres was verified by cardiac synchronicity of bursts, biphasic activity during Valsalva’s manoeuvre, increases during breath-holding, and no change during tactile or auditory stimulation. Raw MSNA was amplified (Nerve Traffic Analyzer 662C-3, Biomedical Engineering Department, University of Iowa, Iowa City, IA, USA) with a total gain of 100,000, bandpass filtered (0.7-2.0 kHz), and integrated with a 0.1 s time constant.

MSNA, ECG, thoracic impedance signals, and finger blood pressure were analogue-to-digital converted using the WinDAQ system (hardware: DI720, software: WinDAQ Pro+, Dataq Instruments Inc., Akron, OH, USA). We analysed ECG, finger blood pressure, respiration, and MSNA offline using a program based on PV-wave software (Visual Numerics Inc., USA) written by one of the authors (A.D.). MSNA bursts were accepted if the signal-to-noise ratio was greater than 2:1 and burst latency was 1.2–1.6 seconds from the preceding R wave. An experienced investigator verified burst detection results. We determined the following MSNA parameters from the integrated nerve signal: burst frequency, i.e. the number of MSNA bursts per minute (bursts/min), burst incidence, i.e. the number of bursts per 100 heart beats (bursts/100 heart beats), as well as MSNA total activity, i.e. the area under the bursts per minute as arbitrary units per minute (au/min).

We assessed spontaneous baroreflex sensitivity from 5-min resting recordings by relating beat-to-beat ECG RR intervals (RRI) and systolic blood pressure (SBP) readings using the cross-spectral and sequence methods. We calculated the baroreflex gain as the mean magnitude value of the cross-spectral transfer function in the low-frequency range (0.05 – 0.15 Hz, squared coherence value >0.5) and as the mean slope of the linear regression line between related RRI and SBP sequences. Sequences are defined as episodes of at least three heart beats with steadily rising (up-sequences) or falling ramps (down-sequences) of RRI (steps >0 ms) and SBP (steps >0.5 mm Hg). Only slopes with a correlation coefficient >0.85 were accepted. The number of up- and down-sequences found in the 5-min recordings was 28±11 and 29±10, respectively.

## Venous samples

Venous plasma catecholamines and renin were determined by high pressure liquid chromatography followed by electrochemical detection^6^ and with an immunoradiometric kit,^7^ respectively. Dihydroxyphenylglycol (DHPG) levels were assessed using gas chromatography mass spectrometry as described earlier.^8^

## Sputum induction and processing

For safety reasons lung function was controlled before and after exposure and during sputum induction by measuring forced expiratory volume in the first second (FEV1). Furthermore, all subjects inhaled 200 µg salbutamol prior to sputum induction to induce bronchodilation. A nose clip was applied and subjects inhaled 3 % pyrogen-free hypertonic saline in 3 consecutive 10-min inhalation periods through a mouthpiece. After each period subjects were asked to produce sputum after carefully wiping the nose and rinsing their mouth and throat to minimize sputum contamination. Then, they were asked to expectorate into a sterile pot. The same procedure was repeated with 4 and 5 % hypertonic saline. When FEV1 decreased >20 % or when participants experienced troublesome symptoms, the procedure was stopped and another salbutamol dose was applied.

Sputum was immediately processed: Sputum plugs were selected, pooled, and homogenized with Sputolysin® (Calbiochem, Darmstadt, Germany). After filtration and centrifugation, supernatants were collected and stored at ‑80 °C before biomarker analysis. Cytospins were prepared and a differential cell count was performed by counting at least 400 non-squamous cells.

## Statistics

The Institute of Biostatistics provided a sample size calculation and a randomized sequence allocation. Sample size calculation was based on a type I error of 5 % (two-sided) and a power of 80 %. According to our previous cross-over study^9^ we assumed an MSNA standard deviation of 7 bursts/min. An MSNA difference of 5 bursts/min is considered clinically relevant. Based on these assumptions complete data sets of all three periods had to be gained in 18 subjects.

The order of interventions followed a Latin square design with blocks of three periods and three sequences. The randomisation list was generated according to the institutional standard operating procedure with SAS 9.3 (SAS Institute Inc., Cary, NC, USA) and delivered in advance to the exposure chamber operators. These operators were bound to secrecy, were not in direct contact with participants, and they were not involved in data collection or analysis. Participants were enrolled by the Fraunhofer Institute for Toxicology and Experimental Medicine and allocated sequentially by the exposure chamber operator. All other staff members and the participants remained blinded with regard to the exposure.

In a fixed effects model, MSNA served as the dependent variable, whereas exposure, patient and period were regarded as independent variables. The approach has been implemented using the general linear models procedure (PROC GLM) in SAS. The analysis was restricted to patients who had completed successfully all three periods. As an additional sensitivity analysis and to confirm the results from the ‘completer’ analysis, an analysis including all patients regardless of missing values was carried out. This approach followed the same steps, but used a mixed model with patients assumed to be ‘random’. We prospectively planned to challenge the claim of an exposure effect in case of discrepancies between ‘complete’ and ‘incomplete’ analysis.

#

# Results

## Exercise


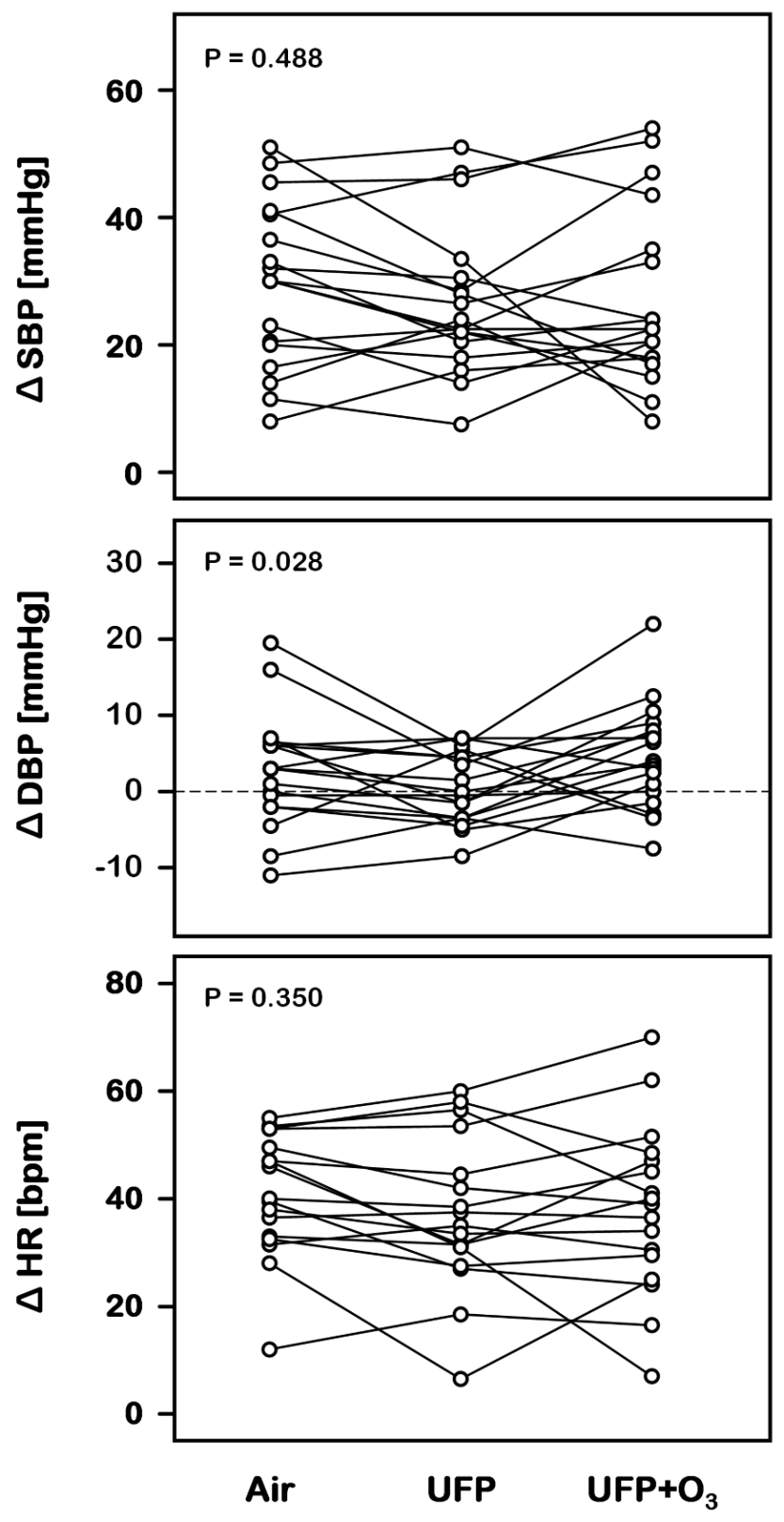


1. Hemodynamic responses to exercise. Individual changes in blood pressure (n=18) and heart rate (n=17) with bicycle exercise in the exposure chamber. Single data points represent the median difference between six 15-min resting and six 15-min exercise periods.
2. Inflammation-related responses to exposure (mean±SD).

| **Parameter** | **Air** | | | **UFP** | | | **UFP+O_3_** | | | ***P* value** |
| --- | --- | --- | --- | --- | --- | --- | --- | --- | --- | --- |
| *Lung function (immediately after exposure vs baseline)* | | | | | | | | | | |
| ΔFVC [ml] | 191 | ± | 180 | 226 | ± | 364 | 8 | ± | 342 | 0.053^†^ |
| ΔFEV1 [ml] | 101 | ± | 88 | 44 | ± | 187 | -57 | ± | 210 | 0.021^‡^ |
| *Blood (~2.5 h after exposure)* | | | | | | | | | | |
| CC16 [µg/l] | 8.7 | ± | 3.7 | 9.0 | ± | 3.7 | 14.4 | ± | 5.4 | 0.000* |
| Leukocytes [Gpt/l] | 8.5 | ± | 2.9 | 7.7 | ± | 2.2 | 10.4 | ± | 3.1 | 0.000* |
| MDA [nM] | 44.5 | ± | 22.0 | 41.4 | ± | 31.1 | 48.3 | ± | 25.9 | 0.780 |
| MPO [µg/l] | 43 | ± | 63 | 29 | ± | 18 | 42 | ± | 38 | 0.248 |
| ShsCRP [mg/l] | 1.7 | ± | 1.4 | 1.5 | ± | 1.1 | 1.7 | ± | 1.8 | 0.845 |
| *Sputum (~3.5 h after exposure)* | | | | | | | | | | |
| Neutrophils [%] | 58 | ± | 14 | 62 | ± | 15 | 82 | ± | 13 | 0.000* |
| MPO: Myeloperoxidase, ΔFVC: Change in forced vital capacity over 3 h of exposure, ΔFEV1: Change in forced expiratory volume in one second over 3 h of exposure, CC16: Club cell secretory protein 16, MDA: Malondialdehyde, ShsCRP: Serum high-sensitivity C-reactive protein.  P values from repeated measures ANOVA.  (*) Air vs. UFP+O_3_ and UFP vs. UFP+O_3_  (†) UFP vs. UFP+O_3_  (‡) Air vs. UFP+O_3_ | | | | | | | | | | |

1. Detailed descriptive statistics for the primary endpoint MSNA [bursts/min] according to the Hills-Armitage approach.^10^

| **Sequence** | **Visit** | **N** | **Mean±SD** | | | **Minimum** | **Maximum** |
| --- | --- | --- | --- | --- | --- | --- | --- |
| ABC | 1 (Air) | 4 | 52.1 | ± | 7.6 | 43.6 | 59.9 |
|  | 2 (UFP) | 4 | 47.6 | ± | 12.4 | 29.0 | 54.7 |
|  | 3 (UFP+O3) | 4 | 47.2 | ± | 17.9 | 30.7 | 69.4 |
| BCA | 1 (UFP) | 7 | 44.9 | ± | 20.3 | 25.4 | 78.6 |
|  | 2 (UFP+O3) | 7 | 41.7 | ± | 13.7 | 25.1 | 60.4 |
|  | 3 (Air) | 7 | 41.3 | ± | 8.2 | 28.2 | 53.5 |
| CAB | 1 (UFP+O3) | 7 | 46.6 | ± | 13.9 | 27.0 | 65.8 |
|  | 2 (Air) | 7 | 50.3 | ± | 16.4 | 35.8 | 85.1 |
|  | 3 (UFP) | 7 | 48.6 | ± | 9.7 | 32.2 | 62.6 |

# References

1. Frampton, M. W. *et al.* Inhalation of ultrafine particles alters blood leukocyte expression of adhesion molecules in humans. *Environ. Health Perspect.* **114,** 51–58 (2006).

2. Frampton, M. W. *et al.* Effects of exposure to ultrafine carbon particles in healthy subjects and subjects with asthma. *Res. Rep. Health Eff. Inst.* 1–47; discussion 49-63 (2004).

3. Elder, A. C., Gelein, R., Finkelstein, J. N., Cox, C. & Oberdorster, G. Pulmonary inflammatory response to inhaled ultrafine particles is modified by age, ozone exposure, and bacterial toxin. *Inhal. Toxicol.* **12 Suppl 4,** 227–246 (2000).

4. Schaumann, F. *et al.* Effects of ultrafine particles on the allergic inflammation in the lung of asthmatics: results of a double-blinded randomized cross-over clinical pilot study. *Part. Fibre Toxicol.* **11,** 39 (2014).

5. Lippmann, M. Health effects of ozone. A critical review. *JAPCA* **39,** 672–695 (1989).

6. Willemsen, J. J., Sweep, C. G., Lenders, J. W. & Ross, H. A. Stability of plasma free metanephrines during collection and storage as assessed by an optimized HPLC method with electrochemical detection. *Clin. Chem.* **49,** 1951–1953 (2003).

7. Danser, A. H. *et al.* Determinants of interindividual variation of renin and prorenin concentrations: evidence for a sexual dimorphism of (pro)renin levels in humans. *J. Hypertens.* **16,** 853–862 (1998).

8. Zoerner, A. A. *et al.* Unique pentafluorobenzylation and collision-induced dissociation for specific and accurate GC-MS/MS quantification of the catecholamine metabolite 3,4-dihydroxyphenylglycol (DHPG) in human urine. *J. Chromatogr. B Analyt. Technol. Biomed. Life Sci.* **879,** 1444–1456 (2011).

9. Tank, J. *et al.* Effect of acute ozone induced airway inflammation on human sympathetic nerve traffic: a randomized, placebo controlled, crossover study. *PLoS One* **6,** e18737 (2011).

10. Hills, M. & Armitage, P. The two-period cross-over clinical trial. *Br. J. Clin. Pharmacol.* **8,** 7–20 (1979).
